# RNA interaction format: a general data format for RNA interactions

**DOI:** 10.1093/bioinformatics/btad665

**Published:** 2023-11-07

**Authors:** Richard A Schäfer, Dominik Rabsch, Guillaume E Scholz, Peter F Stadler, Wolfgang R Hess, Rolf Backofen, Jörg Fallmann, Björn Voß

**Affiliations:** RNA-Biology and Bioinformatics, Institute of Biomedical Genetics, University of Stuttgart, Allmandring 31, 70569 Stuttgart, Germany; Bioinformatics Group, Department of Computer Science, University of Freiburg, Georges-Köhler-Allee 106, 79110 Freiburg, Germany; Bioinformatics Group, Department of Computer Science and Interdisciplinary Center for Bioinformatics, University of Leipzig, Härtelstr. 16-18, 04107 Leipzig, Germany; Bioinformatics Group, Department of Computer Science and Interdisciplinary Center for Bioinformatics, University of Leipzig, Härtelstr. 16-18, 04107 Leipzig, Germany; Genetics and Experimental Bioinformatics, Institute of Biology III, University of Freiburg, Schänzlestr. 1, 79104 Freiburg, Germany; Bioinformatics Group, Department of Computer Science, University of Freiburg, Georges-Köhler-Allee 106, 79110 Freiburg, Germany; Bioinformatics Group, Department of Computer Science and Interdisciplinary Center for Bioinformatics, University of Leipzig, Härtelstr. 16-18, 04107 Leipzig, Germany; RNA-Biology and Bioinformatics, Institute of Biomedical Genetics, University of Stuttgart, Allmandring 31, 70569 Stuttgart, Germany

## Abstract

**Summary:**

RNA molecules play crucial roles in various biological processes. They mediate their function mainly by interacting with other RNAs or proteins. At present, information about these interactions is distributed over different resources, often providing the data in simple tab-delimited formats that differ between the databases. There is no standardized data format that can capture the nature of all these different interactions in detail.

**Availability and implementation:**

Here, we propose the *RNA interaction format* (RIF) for the detailed representation of RNA–RNA and RNA–Protein interactions and provide reference implementations in C/C++, Python, and JavaScript. RIF is released under licence GNU General Public License version 3 (GNU GPLv3) and is available on https://github.com/RNABioInfo/rna-interaction-format.

## 1 Introduction

RNAs have important functions in numerous biological processes, and they typically exert them through direct interaction with other RNAs or proteins. Although there are several databases to collect RNA-based interactions, e.g. sRNATarBase (Wang *et al.* 2016), miRTarBase (Huang *et al.* 2020), snoDB (Bouchard-Bourelle *et al.* 2020), RISE (Gong *et al.* 2018), and sInterBase (Cohen *et al.* 2023), they do not offer a common data format. Hence, data have to be transformed whenever different sources are used. Interactions form networks, therefore data formats for describing networks seem readily applicable. In principle, these resources provide web front-ends which allow to browse its content, but lack any *Application Programmable Interface* (API) to conveniently integrate the data into bioinformatics analyses. As a consequence, the data need to be downloaded in a non-standardized form, such as in tab-delimited text format, which differs for each database. A detailed description and comparison of existing databases and formats are given in the Supplementary Data (text body and Supplementary Tables S1–S3). To collect publicly available RNA interaction data, datasets need to be synchronized and reformatted which can be time-consuming. In that regard, having a common data format ensures interoperability between different resources. For example, the aforementioned databases store somewhat similar information but in different data fields which makes it hard to unify the data (Supplementary Table S2). As of today, multiple data formats are available to describe biological networks. Among them are Extensible Markup Language (XML)-based formats, such as the *Systems Biology Markup Language* (SBML; Hucka *et al.* 2018) to describe biochemical reaction networks. In principle, the *reaction* component in SBML can represent molecular interactions, but its attributes are limited to reaction-specific properties. In addition, the XML format (Bray *et al.* 2018) is verbose as the attributes require opening and closing tags. Although this can catch common errors and incorrect nesting, it introduced overhead which might be problematic in large interaction data. In that regard, XML works with structured data but does not support arrays which further adds to the storage costs of the data. Other XML-based formats which can represent biological networks include the *Proteomics Standard Initiative Molecular Interactions* interchange format (PSI-MI; Hermjakob *et al.* 2004), the *Chemical Markup Language* (CML; Murray-Rust *et al.* 2001), BioPAX (Demir *et al.* 2010), and GraphML (Brandes *et al.* 2002). In addition, other formats are available that simply describe the network but do not capture additional information. These include the *Simple Interaction Format* (SIF), initially developed for Cytoscape (Shannon *et al.* 2003) and the *Nested Network Format* (NNF) that additionally allows to nest subnetworks for a node. In contrast, the *Graph Modelling Language* (GML) consists of hierarchically organized key-value pairs and allows to represent arbitrary data structures in which additional information can be attached to every object. In terms of biological networks, graphs are represented using the node and edge attributes. The JavaScript Object Notation (JSON) is a universal data interchange format that has been widely used in different applications and can also be used to describe graphs. It is human-readable and uses key/value pairs and arrays in JavaScript syntax to describe the data. It is language-independent and can be easily parsed in multiple programming languages. In recent years, there have been efforts to create a JSON graph specification to standardize the description of graphs using the JSON Schema. For that, the JSON Graph Format (JGF) (https://jsongraphformat.info/) has been introduced. At its core is a graph object that represents single or multiple conceptual graphs and contains arbitrary objects at the nodes and edges. It has certain pre-defined key/value pairs, but also allows to specify user-defined values, thereby allowing the specification of metadata objects. This makes it the most comprehensive data format that is able to capture graphs in full. However, JGF describes a specific graph structure that is not able to represent all properties of RNA interactions. Here, we present RNA interaction format (RIF), which is a general JSON-based RNA-RNA/Protein interaction format.

## 2 Format definition

The design of RIF follows some simple rules that should ascertain unambiguous parsability, portability, flexible use with different types of biological molecules, a well-defined minimum amount of mandatory information, handling of user-defined auxiliary information, simple extensibility, and the possibility to validate format compliance. Therefore, we chose JSON, which is built on concepts that are realized in various programming languages. These include key-value pairs (also known as dictionaries, hash tables, or associate arrays) and lists of ordered values (also known as array, vector or list). A RIF file is composed of a list of interaction objects that, in turn, consist of six top-level elements: ID, version, class, type, evidence, and partners. Details about the elements and their mandatory properties are summarized in Table 1 and described in the following. A minimal example is given in Listing 1.

**Table 1.** Overview of the elements of an interaction and their contents in the RIF format.

Element	Value	Description
ID	String	Unique identifier of the interaction
Version	String	Used version of RIF
Class	String	Class of the interaction
Type	String	Chemical nature of the interaction
Evidence	List of objects	Data supporting the interaction
Partners	List of objects	Molecules interacting

**Listing 1.**
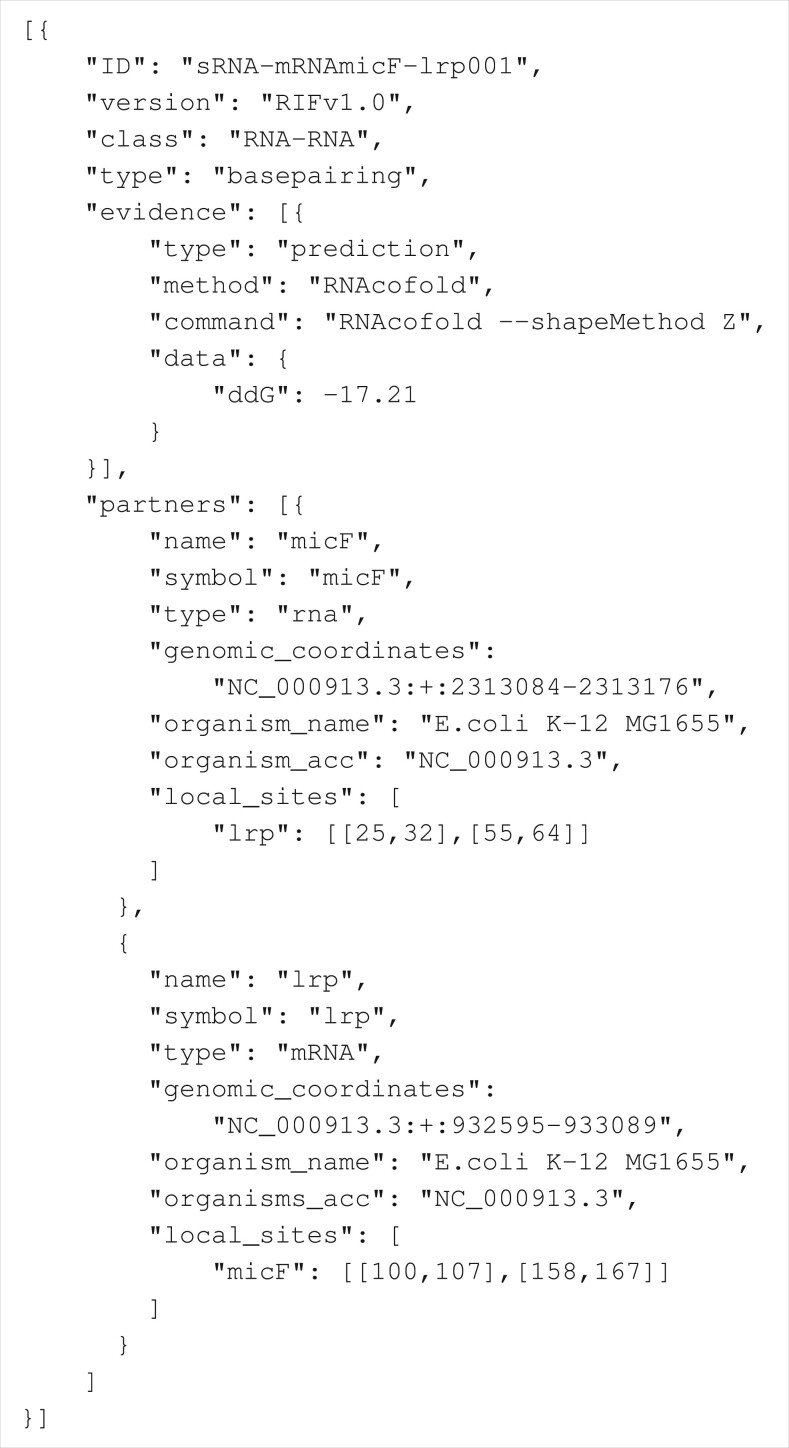
Minimal example of a RIF file.

### 2.1 ID, Version, class, type

These key-value pairs are mandatory and contain general information about the interaction. ID is the identifier of the interaction within the data file. This enables direct access to a specific interaction and has been implemented as a string to ensure the most flexibility. The version attribute of type string describes the used version of the data format. In future versions, this allows for combining different versions of RIF in a single interaction file. The class attribute describes the interaction complex that can either be *RNA–RNA*, *RNA–Protein*, or *Protein–RNA–RNA*. The type of interaction, i.e. the molecular mechanism, is given in the type property. For example, this includes ‘base-pairing’, ‘hydrophobic’, ‘electrostatic’, or any other user-defined type.

### 2.2 Evidence

An interaction object should contain at least one evidence object. Therefore, the key-value pair evidence is an ordered list of evidence objects, which consist of mandatory key-value pairs type, method, and data that describe the supporting evidence for the interaction. The type key-value pair describes the type of evidence and can be an arbitrary term, but should be descriptive, e.g. target prediction, pull-down assay, overexpression. The method attribute declares the technique by which the supporting evidence for the interaction has been generated. This can either be a computational tools such as RNAcofold (Bernhart *et al.* 2006) or experimental techniques, e.g. qPCR. In the former, the optional key-value pair command specifies the command line call. Moreover, the key-value pair data contains user-defined measurements or resources associated with the evidence. In principle, the properties in data can be defined by the user, but the nesting is limited to one additional layer.

### 2.3 Partners

The key-value pair partners is a list of elements that correspond to the RNAs/Proteins involved in the interaction. These contain the mandatory key-value pairs name, symbol, type, and local_sites. The name corresponds to the principal name of the protein/gene/transcript and symbol corresponds to its scientific name. However, this may include user-defined terms, e.g. for newly found transcripts. The attribute type gives the type of the interaction partner as specified in the sequence ontology (Eilbeck *et al.* 2005) and ideally matches the entries in the corresponding annotation file. The genomic_coordinates key-value pair holds the coordinates of the transcript in the genome in the format ’chromosome: strand: start-end’. Attributes organism_name and organism_acc store the organisms scientific name and the accession number of the genome sequence, respectively. The local_sites key-value pair allows to specify the interaction sites on the nucleotide level. It is an ordered list of interaction sites between the partner objects. This means that the first interaction site of the first partner interacts with the first interaction site of the second partner and so on. Each interaction site element in turn is a two-element list specifying start and end of the site. This corresponds to the genomic_coordinates if available. Moreover, info is a nested name/value pair that determines optional properties of the interaction partner. These include the name/value pairs description, sequence and structure. Arbirtrary name-value pairs can be specified as well.

## 3 Implementations and features

We created reference implementations for C/C++, Python and JavaScript and provide a JSON schema based on draft 2020–12. In all, we implemented the functionality to read and write RIF files, to validate the data against the introduced schema, and to retrieve or modify interactions. In addition, the interactions stored in RIF can be exported as Browser Extensible Data (BED) format (https://github.com/samtools/hts-specs) and then visualized in standard genome browsers.

## 4 Discussion

Here, we present a general-purpose interaction format for describing RNA–RNA/Protein interactions, termed RIF, and provide reference implementations for common programming languages. RIF allows to define RNA interaction networks, and simplifies the integration from multiple sources of interaction data. For example, computational tools such as RNAnue (Schäfer and Voß 2021), GraphProt (Maticzka *et al.* 2014; Uhl *et al.* 2021), and PEPPI (Bell *et al.* 2022) can be employed to generate RNA-centric interaction networks from RNA–RNA, RNA–Protein, and Protein–Protein interaction data, respectively.

Furthermore, by design the format should be easy to adapt or to extend to new types of interactions and new classes of biological molecules.

## Supplementary Material

btad665_Supplementary_Data
