# Supplementary material for: RNA interaction format: a general data format for RNA interactions
Source: Bioinformatics. 2023 Nov 7;39(11):btad665. doi: 10.1093/bioinformatics/btad665 (PMC10640394; doi:10.1093/bioinformatics/btad665)
Supplement: btad665_Supplementary_Data [file btad665_supplementary_data.pdf]

SUPPLEMENTARY INFORMATION

RNA Interaction Format - a general data format for RNA interactions

Richard. A. Schäfer,[1] Dominik Rabsch,[4] Guillaume E. Scholz,[2] Peter F. Stadler,[2] Wolfgang R. Hess,[3] Rolf Backofen,[4] Jörg Fallmann[2*] and Björn Voß[1*]

[1]RNA-Biology and Bioinformatics, Institute of Biomedical Genetics, University of Stuttgart, Stuttgart 70569, Germany, [2]Bioinformatics Group, Department of Computer Science and Interdisciplinary Center for Bioinformatics, University of Leipzig, Leipzig 04107, Germany, [3]Genetics and Experimental Bioinformatics, Institute of Biology III, University of Freiburg, Freiburg 79104, Germany and [4]Bioinformatics Group, Department of Computer Science, University of Freiburg, Freiburg 79110, Germany

*Corresponding authors. bjoern.voss@ibmg.uni-stuttgart.de fall@bioinf.uni-leipzig.de

Databases for RNA-RNA/Protein interactions

We examined the formats of available RNA-RNA/Protein interaction databases including **RNAinter** v4.0 (Kang *et al.*, 2022), **miRTarBase** v9.0 (Huang *et al.*, 2022), **sInterBase** (Cohen *et al.*, 2023), **snoDB** v2.0 (Bergeron *et al.*, 2023), **STRING** (Mering *et al.*, 2003). It is to be noted that we focused on the exported data, and the online resources are often more comprehensive. As summarised in Table S1, most commonly, tab-separated values (TSV) or comma-separated values (CSV) are used. In the **STRING** database, the interaction data can be exported as whitespace-separated text files with different granularities, which are accompanied with accessory data. In addition, the whole database can be exported as a SQL dump. Only **RNAinter** provides an *application programming interface* (API), but at the time of writing this manuscript it could not be reached. The records can be queried using different fields. Table S2 lists the information stored in each database. In all databases, specific identifiers which are either common integers (**snoDB**, **sInterBase**) or strings consisting of letters and numbers (**miRTarBase**, **RNAinter**) point to an entry. The symbol/names of the interaction partners are given in all databases, accompanied with the accession identifiers (IDs) when available. For example, **sInterBase** provides the accession ID for the query sRNAs and target mRNAs from EcoCyc (Keseler *et al.*, 2005). More comprehensively, **snoDB** provides accession IDs to HGNC (Seal *et al.*, 2023), RNAcentral (The RNAcentral Consortium, 2019), Ensembl (Martin *et al.*, 2023), RefSeq (O'Leary *et al.*, 2016), Rfam (Kalvari *et al.*, 2021), snoRNABase (Lestrade and Weber, 2006), snoRNA atlas, and snOPY (Yoshihama *et al.*, 2013). In contrast, **RNAinter** and **miRTarBase** do not provide any additional IDs in the exported format, but the online entries link to public databases. Other information about the interaction site such as the genomic coordinates are only provided by **snoDB**. In that regard, the biotype is not listed in **sInterBase** and **miRTarBase**, as these databases mainly contain mRNA targets for sRNAs and miRNAs, respectively. Moreover, the exported formats contain information about the supporting evidence. In **sInterBase** this only consists of the source of the interaction, which can be a study or other database (e.g., **sRNATarBase**). Similarly, **miRTarBase** lists the conducted methodology that supports the interaction (e.g., western blot, qRT-PCR) and links to the study in Pubmed if available. In addition, the supporting evidence is classified into 'weak support' when the evidence is not substantial. Similary, **RNAinter** provides a confidence score that distinguishes between weak (e.g., ChIP-seq and CLIP-seq) and strong (e.g., RNA immunoprecipitation and luciferase reporter assay) evidence. In addition, the prediction method that supports the interaction is also listed. In contrast, **snoDB** exports no concrete evidence that supports the interaction.

Table S1. Overview of the different databases examined in this study.

database	type	search/filter by	format	API
RNAinter v4.0	DNA-RNA, RNA-RNA, RNA-Protein	name/symbol, biotype, species	text file (TSV)	unreachable
miRTarBase v9.0	RNA-RNA	name/symbol, method, literature	excel spreadsheet (xlsx)	no
sInterBase	RNA-Protein	any	text file (CSV)	no
snoDB	RNA-RNA	name/symbol, keywords, box	text file (TSV)	no
STRING	Protein-Protein	name/symbol, species, sequence, arbitrary value/rank	text files, SQL	no

Table S2. Features in the export format of the considered databases.

database	ID	name/ symbol	feature	species	biotype	scoring	sites	experiment	prediction	source/ reference
RNAinter	✓	✓	✓	✓	✓	✓	✗	✓	✓	✗
miRTarBase	✓	✓	✗	✓	✗	✗	✗	✓	✗	✓
sInterBase	✓	✓	✗	✓	✗	✗	✗	✗	✓	✓
snoDB	✓	✓	✓	✓	✓	✗	✗	✗	✗	✗

Data Formats

We examined the available file formats for RNA-RNA/Protein interactions to store interaction data from miRTarBase. In particular, we exported the data associated with the miRTarBase ID MIRT000021 (Table S3). In addition, the online entry contains addtional information of the interaction sites, genomic coordinates, and sequence that were also used. In the following, we describe this interaction using different data formats. It is to be noted that the sequence/structure of TP53INP1 has been truncated. In the following, the interaction MIRT000021 is

Table S3. Exported data of MIRT000021 from MiRTarBase

miRTarBase ID	miRNA	Species (miRNA)	Target Gene	Target Gene (Entrez ID)	Species (Target Gene)	Experiments	Support Type	References (PMID)
MIRT000021	hsa-miR-93-5p	Homo sapiens	TP53INP1	94241	Homo sapiens	Luciferase reporter assay, Western blot	Functional MTI	18974142

described using different data formats. SBML requires 3607 characters to depict MIRT000021. In contrast, RIF only requires 2273 characters, thereby decreasing the filesize by 37%. In SBML, the closing tags mainly contribute to this and generate overhead when parsing large interaction networks. In addition, the absence of arrays/lists in SBML requires additional attributes to store similar data points (e.g., evidence1, evidence2). However, an array package is currently under development and may be available in future versions. Attributes are not restricted and can be defined on any level, which inflates the format and prevents uniform use.

SBML

```
<?xml version="1.0" encoding="UTF-8"?>
<sbml xmlns="http://www.sbml.org/sbml/level3/version1/core" level="3" version="1">
    <model id="RNA-RNA_interaction">
        <listOfCompartments>
            <compartment id="GCF_000001405.40" name="homo_sapiens" size="1" spatialDimensions="3">
                <class>RNA-RNA</class>
                <type>basepairing</type>
                <evidence>
                    <evidence1>
                        <type>experimental</type>
                        <method>Dual luciferase reporter assay</method>
                        <data>
                            <URI>https://doi.org/10.1158/0008-5472.CAN-08-0769</URI>
                            <note>
                                The dual luciferase reporter gene system validated the relationship between hsa-miR-93-5p and TP53INP1
                            </note>
                        </data>
                    </evidence1>
                    <evidence2>
                        <type>experimental</type>
                        <method>Western blot</method>
                        <data>
                            <URI>https://doi.org/10.1158/0008-5472.CAN-08-0769</URI>
                            <note>Western blot analysis detected endogenous TP53INP1 expression upon knockdown of miR-93</note>
                        </data>
                    </evidence2>
                </evidence>
            </compartment>
        </listOfCompartments>
```

```
        <listOfSpecies>
            <species id="hsa-miR-93" name="hsa-miR-93" compartment="GCF_000001405.40">
                <type>rna</type>
                <sequence_type>miRNA</sequence_type>
                <genomic_coordinates>NC_000007.14:+:100093768-100093847</genomic_coordinates>
                <local_sites>
                    <site1>
                        <species>TP53INP1</species>
                        <start>11</start>
                        <end>33</end>
                    </site1>
                </local_sites>
                <info>
                    <sequence>CUGGGGGCUCCAAAGUGCUGUUCGUGCAGGUAGUGUGAUUACCCAACCUACUGCUGAGCUAGCACUUCCCGAGCCCCCGG</sequence>
                    <structure>(((((((((..(((((((((((.((((.(((.((........))))))))))))).))))))).. .)))))))))</structure>
                    <note>accession: MI0000095</note>
                </info>
            </species>
            <species id="TP53INP1" name="TP53INP1" compartment="GCF_000001405.40">
                <type>rna</type>
                <sequence_type>mRNA</sequence_type>
                <genomic_coordinates>NC_000913.3:-:2025223-2025313</genomic_coordinates>
                <local_sites>
                    <site1>
                        <species>hsa-miR-93</species>
                        <start>4617</start>
                        <end>4640</end>
                    </site1>
                </local_sites>
                <info>
                    <description>tumor protein p53 inducible nuclear protein 1</description>
                    <sequence>GTTGCGAGCGCTGCACAACAACAAAAGGACTTGGACTGGCCGGCCTGGGCGCAGCGACCCGAGGGCTGGA...</sequence>
                    <structure>(((((..(((((((((........((((((((((.(((.(((.((((((...)).)))).(((((()).. .</structure>
                </info>
            </species>
        </listOfSpecies>
        <listOfReactions>
            <reaction id="MIRT000021">
                <listOfReactants>
                    <speciesReference species="hsa-miR-93" constant="true"/>
                    <speciesReference species="TP53INP1" constant="true"/>
                </listOfReactants>
            </reaction>
        </listOfReactions>
    </model>
</sbml>
```


RIF


```
[{
    "version": "RIFv1.0",
    "ID": "MIRT000021",
    "class": "RNA-RNA",
    "type": "basepairing",
    "evidence": [
        {
            "type": "experimental",
            "method": "Dual luciferase reporter assay",
            "data": {
                "URI": "https://doi.org/10.1158/0008-5472.CAN-08-0769",
                "note": "The dual luciferase reporter gene system validated the relationship between hsa-miR-93-5p and TP53INP1"
            }
        },
    ],
```

```
    "partners": [
        {
            "name": "hsa-miR-93",
            "symbol": "hsa-miR-93",
            "type": "rna",
            "sequence_type": "miRNA",
            "genomic_coordinates": "NC_000007.14:+:100093768-100093847",
            "organism_name": "Homo sapiens",
            "organism_acc": "GCF_000001405.40",
            "local_sites": {
                "TP53INP1": [[11,33]]
            },
            "info": {
                "sequence": "CUGGGGGCUCCAAAGUGCUGUUCGUGCAGGUAGUGUGAUUACCCAACCUACUGCUGAGCUAGCACUUCCCGAGCCCCCGG",
                "structure": "(((((((((..(((((((((((.((((.(((.((........))))))))))))).)))))))...)))))))))",
                "note": "accession: MI0000095"
            }
        },
        {
            "name": "TP53INP1",
            "symbol": "TP53INP1",
            "type": "mRNA",
            "sequence_type": "rna",
            "genomic_coordinates": "NC_000913.3:-:2025223-2025313",
            "organism_name": "Escherichia coli K-12 MG1655",
            "organism_acc": "NC_000913.3",
            "local_sites": {
                "hsa-miR-93-5p": [[4617,4640]],
            },
            "info": {
                "description": "tumor protein p53 inducible nuclear protein 1",
                "sequence": "GTTGCGAGCGCTGCACAACAACAAAAGGACTTGGACTGGCCGGCCTGGGCGCAGCGACCCGAGGGCTGGA...",
                "structure": "(((((..(((((((((........((((((((((.(((.(((.((((((...)).)))).((((()))..."
            },
        }
    ]
}]
```


References

Bergeron, D. *et al.* (2023). snoDB 2.0: an enhanced interactive database, specializing in human snoRNAs. *Nucleic Acids Res.*, **51**(D1), D291–D296.

Cohen, S. *et al.* (2023). sInterBase: a comprehensive database of Escherichia coli sRNA–mRNA interactions. *Bioinformatics*, **39**(4), btad172.

Huang, H.-Y. *et al.* (2022). miRTarBase update 2022: an informative resource for experimentally validated miRNA–target interactions. *Nucleic Acids Res.*, **50**(D1), D222–D230.

Kalvari, I. *et al.* (2021). Rfam 14: expanded coverage of metagenomic, viral and microRNA families. *Nucleic Acids Res.*, **49**(D1), D192–D200.

Kang, J. *et al.* (2022). RNAInter v4.0: RNA interactome repository with redefined confidence scoring system and improved accessibility. *Nucleic Acids Res.*, **50**(D1), D326–D332.

Keseler, I. M. *et al.* (2005). EcoCyc: a comprehensive database resource for Escherichia coli. *Nucleic Acids Res.*, **33**(suppl_1), D334–D337.

Lestrade, L. and Weber, M. J. (2006). snoRNA-LBME-db, a comprehensive database of human H/ACA and C/D box snoRNAs. *Nucleic Acids Res.*, **34**(suppl_1), D158–D162.

Martin, F. J. *et al.* (2023). Ensembl 2023. *Nucleic Acids Res.*, **51**(D1), D933–D941.

Mering, C. v. *et al.* (2003). STRING: a database of predicted functional associations between proteins. *Nucleic Acids Res.*, **31**(1), 258–261.

O'Leary, N. A. *et al.* (2016). Reference sequence (RefSeq) database at NCBI: current status, taxonomic expansion, and functional annotation. *Nucleic Acids Res.*, **44**(D1), D733–D745.

Seal, R. L. *et al.* (2023). Genenames.org: the HGNC resources in 2023. *Nucleic Acids Res.*, **51**(D1), D1003–D1009.

The RNAcentral Consortium (2019). RNAcentral: a hub of information for non-coding RNA sequences. *Nucleic Acids Res.*, **47**(D1), D221–D229.

Yoshihama, M. *et al.* (2013). snOPY: a small nucleolar RNA orthological gene database. *BMC Res. Notes*, **6**(1), 1–5.
